# Symptomatic loosening of a total knee arthroplasty caused by a tibial chondrosarcoma – a case report

**DOI:** 10.1186/2193-1801-3-308

**Published:** 2014-06-24

**Authors:** Jakob T Sieker, Maximilian Rudert, Andre F Steinert

**Affiliations:** Department of Orthopaedic Surgery, König-Ludwig-Haus, Julius-Maximilians-University Würzburg, Brettreichstr. 11, Würzburg, D-97074 Germany

**Keywords:** Total knee arthroplasty, Bone tumor, Chondrosarcoma, Aseptic loosening

## Abstract

Premature implant loosening following total knee arthroplasty (TKA) can have several causes. In this article we report on a rare case of a 74 year old male patient suffering tibial component loosening 14 month after primary TKA. The patient did neither have any malignancies nor joint arthroplasty before. Upon clinical examination the range of motion in the diseased knee was painfully restricted to 80° of knee flexion, with the patient increasingly suffering sleeping and resting pain, and also at weight bearing. In standard radiographs, loosening of the TKA due to a large osteolysis at the tibial component was evident. Local computed tomography (CT) of the right knee revealed loosening of the tibial component due to a presumably malign bone tumor. For determination of the final diagnosis a representative biopsy of the tumor was taken by open surgery prior to the tumor resection. Histopathologic evaluation of the biopsy revealed a periprosthetic myxoid chondrosarcoma of the proximal tibia. Pre-operative staging examination included CT scans of lung and abdomen, as well as a bone scintigraphy which revealed no signs of tumor metastasis in the body. Surgical management comprised wide tumor resection and implantation of a hinged tumor knee arthroplasty with replacements of the distal femur and proximal tibia, as well as a patella tendon replacement using a synthetic ligament. Revision surgery was necessary twice due to impaired wound healing and critical soft tissue coverage, and treatment included a gastrocnemius muscle flap with skin mesh graft covering. Unfortunately long-term follow-up examinations could not be obtained, as the patient deceased due to an alveolitis during rehabilitation. In summary, the specifics of this rare case of aseptic TKA loosening, and the unusual circumstances of chondrosarcoma diagnosis and treatment are informative for those providing surgical treatment of similar cases.

## Introduction

Chondrosarcoma is an invasive musculoskeletal neoplasm, which is assumed to originate from malign transformed mesenchymal precursor cells, capable of forming a collagenous matrix (Aigner 2002). According to an analysis from the SEER (Surveillance, Epidemiology and End Results) Database the estimated annual incidence is 1/200000 with a mean age of 55 years (Giuffrida et al. 2009). Most frequently chondrosarcoma is located in the appendicular (44.5%) or axial skeleton (31.1%) (Giuffrida et al. 2009). The usual presentation of bone malignancies includes pain, due to destruction of the bone architecture, swelling and occasionally compression of surrounding structures. When bone malignancy is suspected from clinical presentation and supported by radiographic imaging, referral to a specialized interdisciplinary center for further imaging and biopsy according to the American Joint Committee on Cancer (AJCC) guidelines is recommended (Ofluoglu et al. 2010; Edge et al. 2009).

When specified, the most common histological subtype is the myxoid chondrosarcoma (56%), followed by the mesenchymal (25%), dedifferentiated (10%), juxtacortical (4%), malignant chondroblastoma (4%) and clear cell subtype (2%) (Giuffrida et al. 2009). The survival rates differ between histological subtypes and grading, with a thirty year survival rate of low-grade chondrosarcomas (G1 and G2) being 76%, compared to 50% in high-grade tumors (G3 and G4) (Giuffrida et al. 2009).

The predominant treatment of chondrosarcoma is surgical, with the status of the resection margins (R0, R1 or R2) being related with the rate of local recurrence (Giuffrida et al. 2009; Goda et al. 2011). Though skeletal and non-skeletal chondrosarcomas are described to have a high radiation- and chemotherapy-resistance (Onishi et al. 2011; Moussavi-Harami et al. 2006), clinical trials with successful radiation-treatment (Drilon et al. 2008; Rutz et al. 2008) or case-reports with promising chemotherapy application (Drilon et al. 2008; Dallas et al. 2012; Han et al. 2010) do exist. Here we report the diagnostic and surgical management, as well as the short-term outcome of a 76-year-old patient with tibial chondrosarcoma presenting with symptoms of prosthetic loosening 14 month after primary TKA.

## Case report

**Figure 1:**
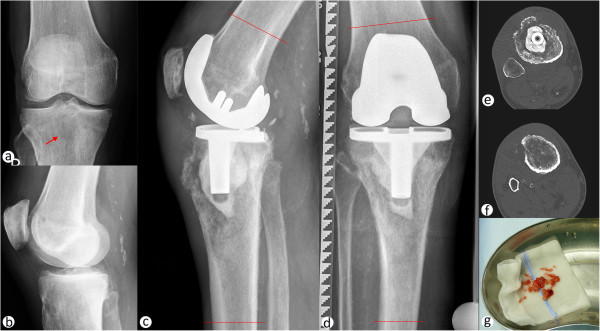
**Implant loosening due to periendoprosthetic tibial chondrosarcoma.** Anterior-posterior **(a)** and lateral **(b)** radiograph prior to total knee arthroplasty (TKA) depicting medial joint space narrowing and meniscal calcification. A radiolucent lesion in the central tibial plateau is indicated by a red arrow. Lateral **(c)** and anterior-posterior radiograph **(d)** 14 month after TKA surgery showing the osteolytic lesion in the proximal tibia, extending through the epi- and metaphyses from the central region to the lateral and anterior cortical bone. Permeation of the cortical bone with discontinuing periosteal reactions, including spiculae formation, can be observed. Preoperative planning was performed to determine plains of resection at 8 cm proximally and 15 cm distally to the joint line (indicated by red lines in c and d). Local CT-scans are showing cortical bone permeation, especially in the anterior and lateral aspects of the proximal tibia at the level of the cemented tibial implant **(e)** and below **(f)**. Open incision biopsy was performed and multiple soft and bone tissue samples were obtained **(g)** and sent for microbiological and histopathological examination.

The patient presented himself in our outpatient clinic 14 month after primary TKA which was implanted due to symptomatic knee osteoarthritis (OA) elsewhere. The pre-operative radiographs with anterior-posterior (ap.) and lateral view of the right knee are shown (Figure 1a and b). After an initial symptom-relief, stress-dependent pain reoccurred few months after the surgery. After a phase of stable symptoms, exacerbation occurred approximately 12 months postoperatively, including resting and sleeping pain, which triggered the referral to our outpatient clinic with suspicion of prosthetic loosening.

In the clinical presentation a painful swelling at the proximal tibia was palpable without signs of inflammation, knee ligaments were stable, while flexion was limited to 80° due to pain. The obtained radiographs with anterior-posterior (ap.) and lateral view of the right knee (Figure 1c and d) showed a suspect Lodwick-grade II osteolytic lesion of the proximal tibia extending from the central epi- and metaphysis to the anterior and lateral cortical bone. The interface to the intact bone was wide and blurred, the cortical bone showed discontinuation and complex reactions including spiculae (Figure 1c and d), which was confirmed by a local CT-scan (Figure 1e and f). In suspicion of bone malignancy, an open incision biopsy was performed that exhibited a whitish tumor mass with myxoid parts (Figure 1g). Histopathological evaluation revealed the diagnosis of a G2 myxoid chondrosarcoma, without signs of bacterial infection.

**Figure 2:**
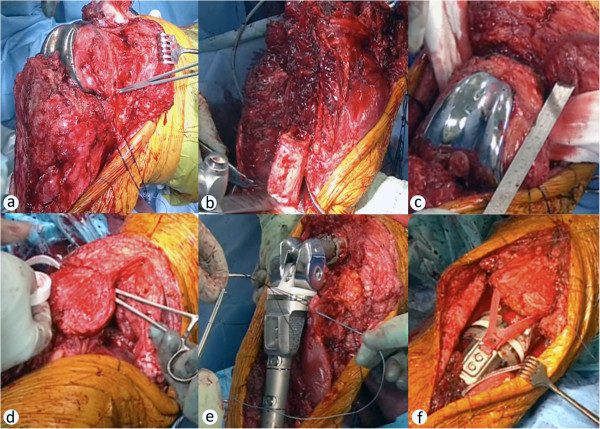
**Surgical management of periendoprosthetic tibial chondrosarcoma.** A medial parapatellar approach was used for exposition, while the patellar-ligament was cut proximally, because of macroscopic suspected tumor infiltration **(a)**. Medial subcutaneous preparation was performed carefully and pes-anserinus muscle insertions were secured for later reattachment **(a)**. After the tibia was exposed 15 cm from joint line, tibial resection was performed as planned and detachment from the popliteal neurovascular structures was performed carefully, allowing en-bloc tumor resection. Because of suspicious marrow tissue a second tibial resection was performed 5 cm more distally **(b)**, which resulted in R0 resection status. The femoral cut was performed as planned at 8 cm proximal to the joint line, to include the femoral component and match the planned femoral component of the revision implant **(c)**. After marrow preparations and trialing, reconstruction of the extensor mechanism was prepared by creating a suprapatellar tunnel in the quadriceps tendon and implantation of an alloplastic ligament **(d)**. Original components were cemented, using the prior tested sizes. Pes-anserinus and biceps-femoris tendons were reattached at the correspondent implant sites **(e)**. The alloplastic ligament was secured with the screw-mechanism of the tibial implant and additional sutures **(f)**. Drains were implanted distant to the tumor location and tension-free wound closure was performed **(f)**.

Staging was then completed and the case was discussed in the local interdisciplinary tumor board. In summary a local disease stage, without infiltration of popliteal structures or metastases was present, and a wide resection of the tumor with inclusion of the knee prosthesis was indicated according to the disease stage. Since limb-preserving resection and reconstruction is technically tedious and carries the risk of incomplete resection and local recurrence, amputation was also discussed with the patient and his family. In consideration of all pros and cons of the respective approaches, the patient wished to undergo wide tumor resection and reconstruction with a distal femur and proximal tibia replacing hinged knee prosthesis. This was then planned using the radiographs and CT-scans (Figure 1c-f), with the projected resections being located 8 cm proximal of the joint line at the femur and 15 cm distally of the joint line at the tibia. For the reconstruction the MML tumor knee system for distal femoral and proximal tibial replacement (Modular endoprosthetic system Munich-Lübeck, Orthodynamics GmbH, Lübeck, Germany) was chosen (Salis-Soglio et al. 2010), with an alloplastic reconstruction of the extensor mechanism (Holzapfel et al. 2011).Resection of the knee joint with proximal tibia: Resection steps included scar excision, resection of the inner part of the patella with complete patellar-tendon (Figure 2a), resection of the joint capsule under preservation of the muscle-insertions of the pes-anserinus (Figure 2a) and the biceps-femoris tendons. Resection was initially performed as previously planned as soft tissue structures were without signs of tumor infiltration. After en-bloc resection the distal marrow showed suspicious tissue, therefore the intraoperative decision to extent the resection 5 cm distally was made (Figure 2b), which resulted in macro- and microscopically tumor free margins. The femoral resection was performed as planned 8 cm proximal of the joint line to match the planned implant, while no suspect bone or soft tissue changes were observed at the resection margins (Figure 2c). All resected tissues were sent in for histopathological examination. For reconstruction medullary canals were prepared followed by implantaion of trial-components for joint-line resonstruction, resulting in full extension and flexion without instability. Thorough irrigation was performed between steps and prior to cement fixation of the original components in the previously tested sizes. After reposition and connection, the previously detached tendons were reattached using FiberWire ® sutures (Arthrex, Naples, FL, USA), with reconstruction of the patellar tendon being performed using a Trevira synthetic ligament (Telos, Marburg, Germany) (Figure 2d-f), before tension free wound closure was performed. After surgery full weight-bearing with an extension brace and continuous passive motion was allowed.

**Figure 3:**
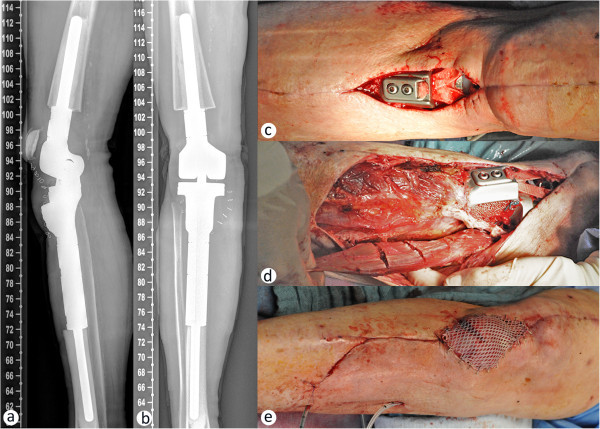
**Soft tissue coverage following megaendoprosthetic knee reconstruction.** Postoperative lateral **(a)** and anterioposterior **(b)** radiographs showing correct implant positioning. Due to the necessity of the patellar-ligament resection, absent soft tissue cover of the proximal anterior portion of the tibial implant caused wound-healing deficiencies, which required operative revisions **(c)**. To achieve soft tissue coverage a medial gastrocnemius muscle flap was prepared **(d)** and covered with a mesh graft from the opposite thigh **(e)**.

The final histopathological examination revealed a pT2 pN0 L0 V0 tumor stage, with proof of the prior G2 grading. Resection status was proven as R0. While the postoperative radiographs (Figure 3a, b) showed correct implant positioning without signs of loosening or fracture, mobilization needed to be restricted due to delayed wound-healing over the site of patellar ligament-reconstruction, due to absent soft-tissue covering. This made operative wound revision necessary, including debridement (Figure 3c), gastrocnemius-flap-reconstruction (Figure 3d) in combination with a mesh-graft (Figure 3e) from the opposite thigh necessary. Thereafter he patient was mobile on the ward stage and healing of the graft was observed, as the patient could be discharged and transferred to a stationary rehabilitation department. Although all intraoperative cultures were sterile, antibiotic prophylaxis was continued orally during rehabilitation. Unfortunately - after initial progress in terms of mobility - the patient developed a progressive respiratory insufficiency due to an alveolitits, and deceased finally after a 4 week course of ICU care. Written consent was obtained from the patient’s wife to publish data and pictures related to the treatment of her husband.

## Discussion

The described case presented with symptomatic loosening 14 months after TKA. Although reports of implant-related sarcoma formation do exist, in these the minimal observed interval from implantation to sarcoma diagnosis was 2.5 years (Keel et al. 2001). Since our case presented with an extensive tumor size early after TKA and an osteolytic lesion was described in the protocol of the externally performed surgery, we suspect that the chondrosarcoma was present during initial surgery. However, a biopsy, obtained from the osteolytic lesion during primary TKA remained inconclusive. Towards the management of patients with an extensive osteolytic lesion in combination with OA, we recommend the open-incision biopsy prior to TKA and the evaluation in a pathological institute with expertise in musculoskeletal malignancies.

Towards the decision between amputation and prosthetic reconstruction, the local tumor extension, especially in relation to the adjacent nerves and vasculature, is important. In the presented case, the vasculature sparing R0 resection was possible and the limb-preservation using a proximal tibia replacing knee prosthesis was performed according to the patient’s wish. For resection, a surgical approach through the patella bone was chosen, comprising the whole knee capsule, patellar and quadriceps tendon, followed by alloplastic reconstruction. Though technically feasible, this procedure obviously carries the risk of soft tissue loss, predisposing for wound-healing problems above the insertion site of the alloplastic graft. In our case, a primary soft tissue coverage using a gastrocnemius-flap-graft maybe would have enabled for faster wound healing and recovery. Therefore, soft tissue muscle flaps should be readily indicated and performed, when soft tissue coverage of large metal implants appears critical or insufficient.

Regrettably, after further progress towards mobility during his stationary rehabilitation, the patient deceased from an alveolitis. Post mortem examination to investigate the etiology of the lung disease was not performed. Therefore this case report is limited to the diagnostic and operative management, as well as the short-term outcome at discharge from our stationary care.

The comparison of outcomes with alternative surgical procedures (endoprosthetic reconstruction versus amputation) is hardly possible, due to the low incidence and different stages of the disease. To date, no clinical trial comparing outcomes of tumor replacing joint arthroplasty and amputation in joint-involving chondrosarcoma does exist to our knowledge. Therefore decision of surgical management is highly individual, primarily depending on disease stage, grade and patient preferences.
